# Blocking *dPerk* in the intestine suppresses neurodegeneration in a *Drosophila* model of Parkinson’s disease

**DOI:** 10.1038/s41419-023-05729-9

**Published:** 2023-03-22

**Authors:** Rebeka Popovic, Amrita Mukherjee, Nuno Santos Leal, Lydia Morris, Yizhou Yu, Samantha H. Y. Loh, L. Miguel Martins

**Affiliations:** MRC Toxicology Unit, University of Cambridge, Cambridge, UK

**Keywords:** Parkinson's disease, Energy metabolism, Cell death in the nervous system, Mitophagy

## Abstract

Parkinson’s disease (PD) is characterised by selective death of dopaminergic (DA) neurons in the midbrain and motor function impairment. Gastrointestinal issues often precede motor deficits in PD, indicating that the gut-brain axis is involved in the pathogenesis of this disease. The features of PD include both mitochondrial dysfunction and activation of the unfolded protein response (UPR) in the endoplasmic reticulum (ER). PINK1 is a mitochondrial kinase involved in the recycling of defective mitochondria, and *PINK1* mutations cause early-onset PD. Like PD patients, *pink1* mutant *Drosophila* show degeneration of DA neurons and intestinal dysfunction. These mutant flies also lack vital proteins due to sustained activation of the kinase R-like endoplasmic reticulum kinase (dPerk), a kinase that induces the UPR. Here, we investigated the role of dPerk in intestinal dysfunction. We showed that intestinal expression of *dPerk* impairs mitochondrial function, induces cell death, and decreases lifespan. We found that suppressing dPerk in the intestine of *pink1*-mutant flies rescues intestinal cell death and is neuroprotective. We conclude that in a fly model of PD, blocking gut-brain transmission of UPR-mediated toxicity, is neuroprotective.

## Introduction

Parkinson’s disease (PD) is a neurodegenerative disease and one of the most common movement disorders. At present, over 10 million people have PD (reviewed in [1]). The motor disturbances experienced by PD patients are linked to the selective loss of dopaminergic (DA) neurons in the substantia nigra pars compacta (SNpc). One of the most common and disabling nonmotor symptoms of PD is constipation, a form of gut impairment [2]. There are substantive data suggesting that alterations in the intestine may precede neurodegeneration in patients with PD (reviewed in [3]). *Postmortem* studies have suggested that α-synuclein aggregates that contribute to PD travel from the enteric nervous system in the gut to the central nervous system and cause disease pathology [4]. Studies in which α-synuclein pathology was modelled in mice showed that gut-to-brain transmission of this toxic protein causes PD-associated neurodegeneration in the CNS [5], supporting the concept that alterations in the gastrointestinal tract can cause neurodegeneration. Mitochondrial dysfunction is a hallmark of PD (reviewed in [6]). Mitochondria accumulate damage with age, and cells have essential quality control mechanisms to ensure mitochondrial health (reviewed in [7]). Damaged mitochondria can be cleared and recycled via mitophagy, a specialised form of autophagy. *PINK1* codes for a mitochondrial kinase that promotes mitophagy and ensures mitochondrial health (reviewed in [6]). Mutations in *PINK1* cause autosomal recessive PD [8], and the fruit fly (*Drosophila melanogaster*) has been used to model *PINK1*-dependent PD. *Pink1* mutant *Drosophila* show degeneration of DA neurons and global activation of R-like endoplasmic reticulum kinase (dPerk) signalling. In *pink1* mutant flies, dPerk activates the unfolded protein response (UPR), leading to shutdown of protein synthesis [9]. Genetic or pharmacological inhibition of dPerk in *pink1* mutants is neuroprotective [9]. The UPR normally acts to protect cells against stress that results from the accumulation of unfolded or damaged proteins. However, several studies indicate that when this pathway becomes constitutively activated, it reduces mRNA translation and promotes neurodegeneration (reviewed in [10]). In *pink1* mutant flies, preventing cell death in the gut blocks the degeneration of DA neurons in the CNS, further highlighting the potential role of gut-brain communication in PD models ([11] and reviewed in [12]). dPerk is also important for intestinal homeostasis [13]. As dPerk also plays a role in neurodegeneration in fly models of PD, we explore the role of this kinase in gut-brain signalling. We show that dPerk expression in the gut causes intestinal dysfunction and that silencing dPerk in the gut protects neurons in the CNS of *pink1* mutant flies. We provide further evidence for gut-brain axis signalling in a model of PD associated with mitochondrial defects.

## Results

### dPerk expression results in intestinal dysfunction and decreased lifespan

**Fig. 1:**
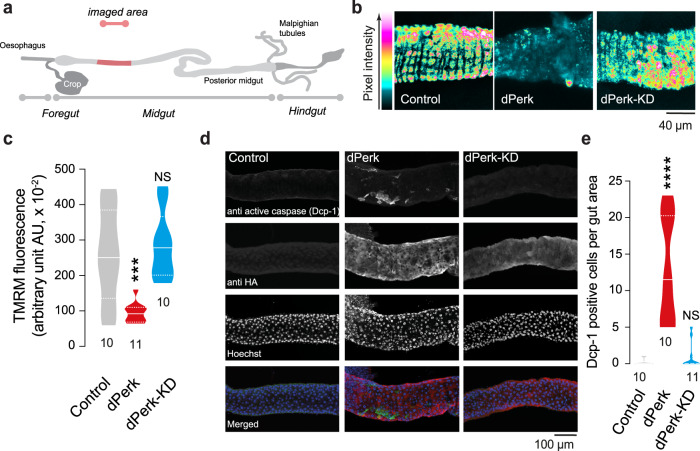
dPerk expression in the gut compromises mitochondrial function and induces cell death. **a** An illustration of the imaged region of the adult fly gut. **b**, **c** Expression of dPerk leads to a reduction in the Δψm in the fly intestine. **b** Representative confocal images showing whole-mounted intestine loaded with TMRM. The intensity of TMRM is visualised using a five-tone heatmap. **c** Quantitative data (asterisks, one-way ANOVA with Dunnett’s multiple comparison test, comparison of control flies with dPerk and dPerk-KD flies, n indicates the number of guts). The data are shown as the average of 8 ROIs per gut. **d**, **e** dPerk expression in the intestine causes caspase (Dcp-1) activation. **d** Representative confocal images showing Dcp-1-positive cells in the midgut. Both dPerk constructs (dPerk and dPerk-KD) were tagged with HA. Green, Dcp-1; red, HA; blue, cell nuclei. **e** Quantification of Dcp-1-positive cells in the midgut (asterisks, Kruskal‒Wallis with Dunn’s multiple comparison test, comparison of control flies with dPerk and dPerk-KD flies, n indicates the number of guts). Data from 10-day-old male flies were analysed. Genotypes: control: *w; NP3048Gal4/* + *;* + , dPerk: *w; NP3048Gal4/UASdPerk*; +, dPerk-KD: *w; NP3048Gal4/UASdPerk-K671R;* +.

**Fig. 2:**
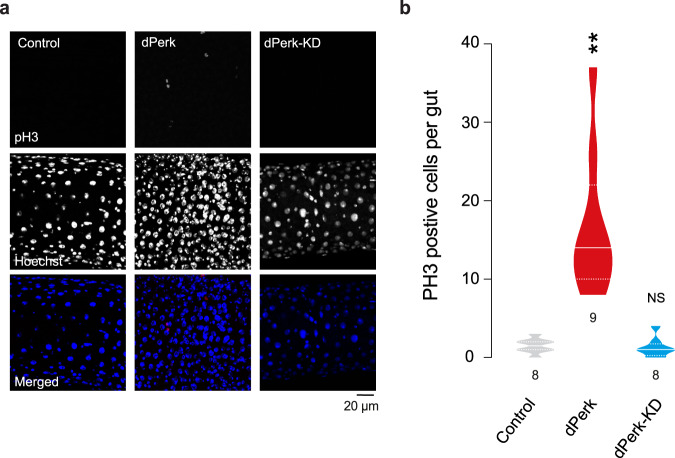
dPerk expression increases cell proliferation in the gut. **a**, **b** Cell proliferation was measured by counting the number of PH3-positive cells. **a** Representative confocal images showing PH3-positive cells in the midgut. Red, PH3; blue, cell nuclei. **b** Quantification of PH3-positive cells in the midgut (asterisks, Kruskal‒Wallis with Dunn’s multiple comparison test, comparison of control flies with dPerk and dPerk-KD flies, *n* indicates the number of guts). The number of PH3-positive cells in the anterior and posterior midgut (from the proventriculus region to the posterior midgut-hindgut junction) was determined. Data from 10-day-old male flies were analysed. Genotypes: control: *w; NP3048Gal4/* + *;* + , dPerk: *w; NP3048Gal4/UASdPerk*; +, dPerk-KD: *w; NP3048Gal4/UASdPerk-K671R;* +.

*Pink1* mutant flies exhibit compromised mitochondrial function, characterised by loss of the mitochondrial membrane potential (Δψm) and activation of the kinase dPerk [9]. They also show intestinal dysfunction characterised by an increase in cell death in the midgut [11]. We first tested whether prolonged expression of dPerk in intestinal cells is sufficient to trigger mitochondrial dysfunction. To assess mitochondrial function in the intestine, we measured Δψm using tetramethylrhodamine methyl ester (TMRM). We observed that the expression of dPerk caused loss of the Δψm in the fly midgut (Fig. 1a–c), indicating that sustained activation of dPerk in the intestine is sufficient to impair mitochondrial function. Mitochondria play a key role in controlling apoptosis in *Drosophila* (reviewed in [14]), and intestinal apoptosis in flies can be inhibited by the expression of Buffy, a member of the Bcl-2 family of apoptosis antagonists [11]. We therefore tested whether dPerk-dependent loss of the Δψm is linked to cellular death in the intestine. We found that the expression of dPerk, but not its kinase-dead counterpart (dPerk-KD), caused a significant increase in the levels of active death caspase-1 (Dcp-1), an effector caspase in *Drosophila* that is important for apoptosis (Fig. 1d, e). Cell death in the gut results in a proliferative response by intestinal stem cells (ISCs) [15]. We next measured the degree of cellular proliferation in dPerk-expressing flies by monitoring the number of phospho-histone H3 (PH3)-positive cells. Histone H3 is phosphorylated during mitotic cell division, and we found that dPerk expression causes an increase in the number of cells positive for this proliferation marker (Fig. 2a, b). Taken together, these data indicate that dPerk expression causes intestinal damage by compromising mitochondrial function and inducing cell death and that the gut then compensates for this loss of cells by increasing proliferation.

**Fig. 3:**
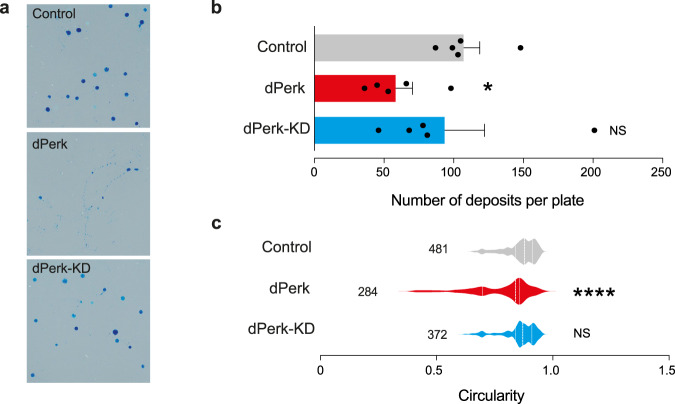
Expression of dPerk in the gut compromises intestinal function. **a** Representative images of fly faeces over a 20-hour feeding period. Data from 10-day-old male flies fed on food supplemented with blue dye to aid the visualisation of the faeces were analysed. **b** Expression of dPerk reduces the number of faecal deposits. The data are shown as the number of deposits per plate (means ± sem, asterisks, Kruskal‒Wallis with Dunn’s multiple comparison test, comparison of control flies with dPerk and dPerk-KD flies). **c** Expression of dPerk alters the shape of faecal deposits (asterisks, Kruskal‒Wallis with Dunn’s multiple comparison test, comparison of control flies with dPerk and dPerk-KD flies, n indicates the number of faecal deposits). Genotypes: control: *w; NP3048Gal4/* + *;* + , dPerk: *w; NP3048Gal4/UASdPerk*; +, dPerk-KD: *w; NP3048Gal4/UASdPerk-K671R;* +.

Next, we assessed the physiological consequences of intestinal damage caused by dPerk expression in the gut. We performed quantitative analysis of fly faecal deposits using the method described by Cognigni and colleagues [16]. dPerk-expressing flies showed a reduction in the number of deposits per plate, suggesting that intestinal dysfunction leads to reduced faecal output (Fig. 3a, b). dPerk expression further leads to a reduction in the circularity of the faeces (Fig. 3a, c). Together, these findings show that dPerk-dependent intestinal damage leads to physiological changes in fly defecation.

**Fig. 4:**
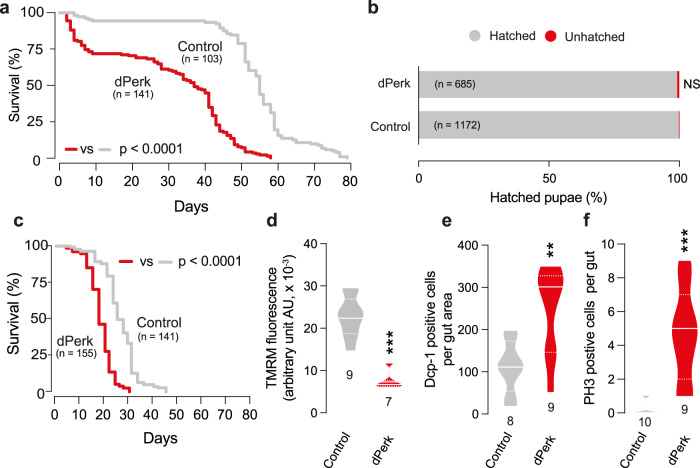
Intestinal dysfunction driven by dPerk decreases lifespan. **a** Lifespan analysis showed that the intestinal expression of dPerk in flies reduces lifespan (asterisks, log-rank test) but **b** does not affect the development of the flies (asterisks Chi-square two-tailed, 95% confidence intervals). Temporal control expression of dPerk using Gal80^ts^ in the flies **c** reduces lifespan (asterisks, log-rank test) and in 13 day old males, **d** decreases Δψm (asterisks, Mann-Whitney two-tailed test), **e** increase caspase (Dcp-1) activation (asterisks, two-tailed Student’s t-test) and **f** increases the number of PH3-positive cells (asterisks, Mann-Whitney two-tailed test) in the fly intestine. Genotypes: **a**, **b** control: *w; NP3048Gal4/* + *;* + , dPerk: *w; NP3048Gal4/UASdPerk*; +, **c**–**f** control: *w; NP3048Gal4/* + *; Gal80*^*ts*^*/+*, dPerk: *w; NP3048Gal4/UASdPerk*; *Gal80*^*ts*^*/+*.

Ageing leads to functional decline of individual organs, such as the gut. In flies, intestinal defects predict age-onset mortality [17]. Therefore, we tested whether dPerk-dependent intestinal toxicity can lead to premature death. Our analysis showed a reduction in the lifespan of flies in which intestinal toxicity was triggered by dPerk expression (Fig. 4a).

The *NP3084*Gal4 driver can induce the expression of dPerk throughout fly development. We therefore examined the potential effect on development of expressing dPerk using this driver, by evaluating the eclosion of adult flies. We observed comparable eclosion rates between control flies and flies expressing dPerk (Fig. 4b) showing that the decrease in fly lifespan following its intestinal upregulation is not due to a developmental abnormality.

Therefore, to further prove that the damaging effects of dPerk observed is not due to expression throughout development, we used the Gal4-UAS gene expression system modified with a temperature-sensitive Gal80 protein (Gal80^ts^), ubiquitously expressed from the tubulin 1*α* promoter [18]. At 18 °C, the ubiquitously produced Gal80 protein is functional and acts as a repressor of the Gal4 transcriptional activator by binding to the Gal4 protein. At restrictive temperatures above 29 °C, Gal80 loses its ability to bind Gal4 and its repressive role, allowing the target gene to express under the control of the upstream activation sequence (UAS). Flies were grown at 18 °C and the eclosed adults were kept at 29 °C from 1 day post-eclosion, allowing for temporally regulated intestinal expression of dPerk in adult flies with the *NP3084*Gal4 driver. We found that the expression of dPerk in the adult intestine alone, caused a decrease in lifespan (Fig. 4c), a decrease in mitochondrial function in the intestinal cells (Fig. 4d), as well as an increase in both intestinal apoptosis (Fig. 4e) and cell proliferation (Fig. 4f).

### Silencing of dPerk suppresses intestinal defects in *pink1* mutant flies

**Fig. 5:**
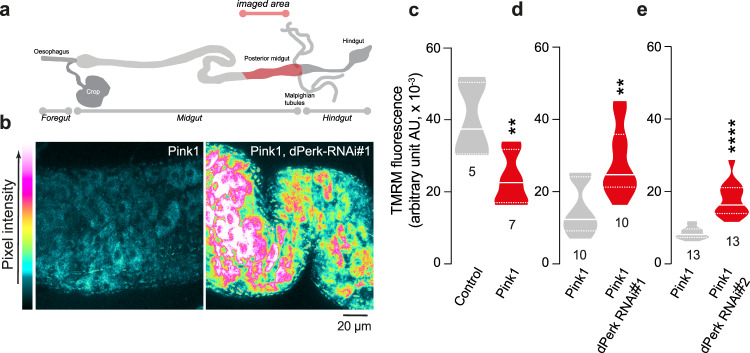
Suppression of dPerk in the midgut of *pink1* mutants rescues mitochondrial dysfunction. **a** An illustration of the imaged region in the posterior midgut of the adult fly. **c** *pink1* mutant flies show loss of the Δψm in the midgut (mean ± sem; asterisks, unpaired t test). **d**–**e** Recovery of the Δψm upon intestinal RNAi-mediated suppression of dPerk, with two independent RNAi lines, in *pink1* mutant flies. **b** Representative confocal images showing the midguts of adult flies loaded with TMRM. The intensity of TMRM is visualised using a five-tone heatmap. Quantitative data (mean ± sem; asterisks, unpaired t test) with RNAi#1 **d** and RNAi#2 **e**. The flies were 10 days old in **b** and 20 days old in **c**–**e**. The data are shown as the average of 8 ROIs per gut. Genotypes: control: *w; NP3048Gal4/* + *;* + , Pink1: *pink1B9; NP3048Gal4/* + ; + , Pink1, dPerk-RNAi#1: *pink1B9; NP3048Gal4/UAS dPerk RNAi#1*; +, Pink1, dPerk-RNAi#2: *pink1B9; NP3048Gal4/UAS dPerk RNAi#2*; +.

**Fig. 6:**
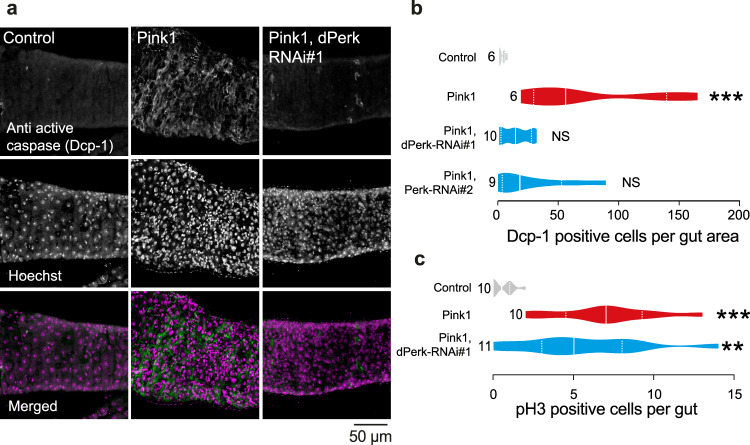
Suppression of dPerk in the midguts of *pink1* mutants rescue intestinal dysfunction. **a**, **b** Caspase activation in the intestine of *pink1* mutant flies was blocked by dPerk RNAi. **a** Representative confocal images showing Dcp-1-positive cells in the posterior midgut region. **b** Quantitation of Dcp-1-positive cells in the posterior midgut showing that silencing dPerk reduced apoptosis of enterocytes of *pink1* mutant flies (asterisks, one-way ANOVA with Dunnett’s multiple comparison test, all genotypes are compared to the control, n indicates the number of guts). **c** The number of PH3-positive cells in *pink1* mutants was not affected by RNAi-mediated suppression of dPerk (asterisks, Kruskal‒Wallis with Dunn’s multiple comparison test, all genotypes are compared to the control, n indicates the number of guts). Data from 30-day-old male flies were analysed. Genotypes: control: *w; NP3048Gal4/* + *;* + , Pink1: *pink1B9; NP3048Gal4/* + ; + , Pink1, dPerk-RNAi#1: *pink1B9; NP3048Gal4/UAS dPerk RNAi#1*; +, Pink1, dPerk-RNAi#2: *pink1B9; NP3048Gal4/UAS dPerk RNAi#2*; +.

Studies on *pink1* mutant *Drosophila*, which are used to model PD, have indicated that mitochondrial dysfunction is an important feature of the pathogenesis of PD (reviewed in [6]). Mitochondrial impairment in *pink1* mutant flies compromises neuronal function. In *pink1* mutant young adults, mitochondrial impairment causes defective secretion of neuropeptides, leading to sleep defects [19]. *Pink1* mutations also cause loss of DA neurons in aged flies [20], which can be prevented by the inhibition of dPerk [9]. *Pink1* mutant flies also show intestinal dysfunction and blocking cell death in the intestine of these flies is neuroprotective [11]. We show here that the expression of dPerk in the gut is sufficient to cause mitochondrial dysfunction (Fig. 1) and compromise gut health. Additionally, suppression of dPerk in *pink1* mutants is neuroprotective [9]. Therefore, we next asked whether suppressing dPerk in the intestine of *pink1* mutant flies is sufficient to restore neuronal heath. We assessed mitochondrial function in the midguts of *pink1* mutant flies (Fig. 5a–c) and observed loss of the Δψm. Next, we suppressed dPerk expression in the gut by RNA interference (RNAi) using the *NP3084* midgut specific *Gal4* driver. Downregulation of dPerk increased the Δψm in the intestine of *pink1* mutant flies (Fig. 5d, e). We confirmed that cell death is increased in *pink1* mutant flies, as previously reported [11], by assessing active Dcp-1 levels. We found that RNAi-mediated downregulation of dPerk suppressed intestinal apoptosis in *pink1* mutants (Fig. 6a, b). We also measured the levels of PH3, a marker for proliferation of intestinal stem cells (ISCs), to assess if the reduction of intestinal cell death modulates the proliferative response in these cells [21]. We confirmed that the number of PH3-positive cells was increased in the *pink1* mutants, as previously reported [11]. However, RNAi-mediated downregulation of dPerk did not affect the number of PH3-positive cells in the guts of these mutants (Fig. 6c).

We conclude that silencing dPerk in the intestine of *pink1* mutant flies restores mitochondrial health and blocks apoptosis in midgut enterocytes, while having a negligible effect on the proliferation of ISCs.

### Suppressing dPerk in the intestine of *pink1* mutant flies is neuroprotective

Constipation, a form of gut dysfunction, is one of the most common and disabling nonmotor symptoms of PD [2].

**Fig. 7:**
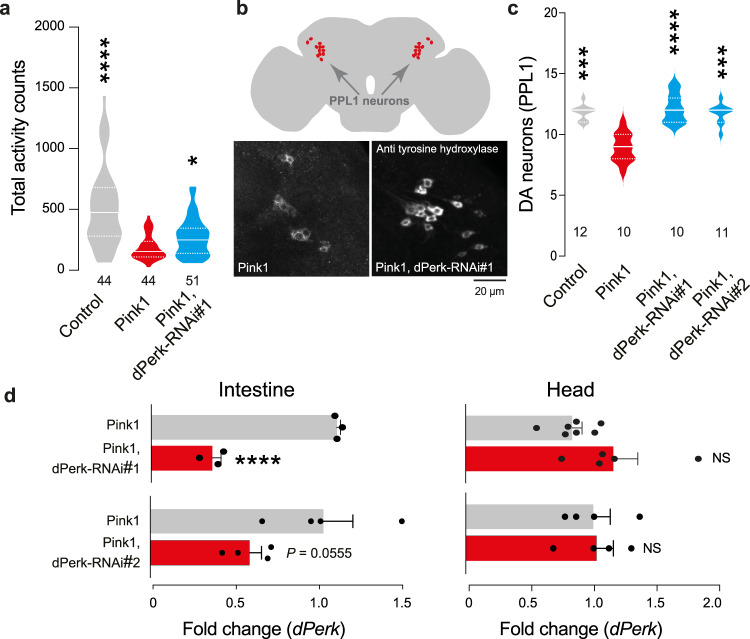
Neuronal defects in *pink1* mutants are rescued by suppression of dPerk in the midgut. **a** Intestinal suppression of dPerk rescued activity defects in *pink1* mutants (asterisks, Kruskal‒Wallis with Dunn’s multiple comparison test, comparison of *pink1* mutant flies with control flies and dPerk-RNAi-treated *pink1* mutant flies, n indicates the number of flies). **b**, **c** Intestinal suppression of dPerk prevented loss of the PPL1 cluster of DA neurons in *pink1* mutants. **b** Top: schematic representation of the PPL1 anatomical location. Bottom: representative confocal images showing loss (and rescue) of PPL1 neurons in *pink1* mutant and dPerk-RNAi-treated *pink1* mutant flies. **c** RNAi-mediated suppression of dPerk in the intestine prevented loss of the PPL1 cluster of DA neurons in *pink1* mutants (asterisks, Kruskal‒Wallis with Dunn’s multiple comparison test, comparison of *pink1* mutant flies with control flies and dPerk-RNAi-treated *pink1* mutant flies, n indicates the number of brains). **d** RNAi-mediated suppression of dPerk using a gut-specific driver reduced the mRNA levels of dPerk in the intestine (left, means ± sem; asterisks, unpaired t test, *n* indicates the number of biological replicates) but not in the heads (right) of *pink1* mutants (means ± sem; NS, unpaired t test, n indicates the number of biological replicates). Data from 30-day-old male flies were analysed. Genotypes: **a–c** control: *w;NP3084Gal4/* + *;* + , Pink1: *pink1B9; NP3048Gal4/* + ; + , Pink1, dPerk-RNAi#1: *pink1B9; NP3048Gal4/UAS dPerk RNAi#1*; +, Pink1, dPerk-RNAi#2: *pink1B9; NP3048Gal4/UAS dPerk RNAi#2*; +.

In *pink1* mutant flies, gut dysfunction is associated with neurodegeneration and sleep defects [11]; therefore, we evaluated whether RNAi-mediated silencing of dPerk in the guts of *pink1* mutant flies can rescue these neuronal defects. Downregulation of dPerk reversed the defects in total activity of *pink1* flies (Fig. 7a). We then examined the effects of dPerk suppression in the intestine on the degeneration of DA neurons in the brains of *pink1* mutant flies. We found that RNAi-mediated suppression of dPerk in the intestine rescued the loss of DA neurons in *pink1* mutants (Fig. 7b, c). To confirm that the *NP3084*Gal4 driver only silenced dPerk in the gut, we measured the transcript levels of *dPerk* in the guts and heads of *pink1* mutant and *pink1* mutant with RNAi suppression of dPerk. The results showed that suppression of dPerk in the guts of *pink1* mutant or *pink1* mutant with RNAi suppression of dPerk, decreased the level of this transcript in the gut but not in the head (Fig. 7d).

**Fig. 8:**
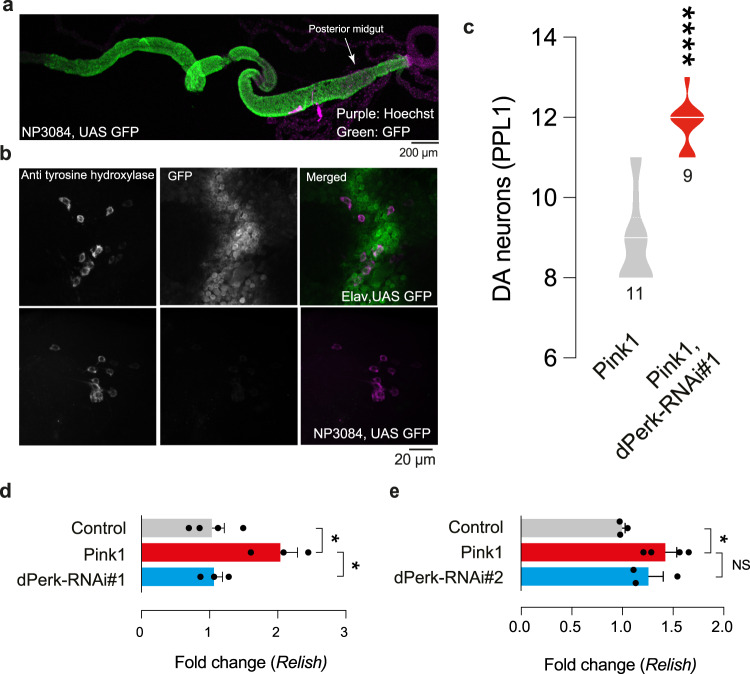
Upregulation of relish in *pink1* mutants are reversed by suppression of dPerk in the midgut. **a** A representative image showing *NP3084Gal4* drives GFP expression throughout the adult fly midgut. **b** *NP3084Gal4* does not drive expression in the PPL1 cluster region of the adult fly brain. We used either the *elav-Gal4* (top) or the *NP3084Gal4* (bottom) to assay GFP fluorescence in adult fly brains in the PPL1 cluster of DA neurons. **c** RNAi-mediated suppression of dPerk in the intestine, using the *mex1Gal4* driver prevented loss of the PPL1 cluster of DA neurons in *pink1* mutants (asterisks, Mann-Whitney two-tailed test, n indicates the number of brains). **d**, **e** Analysis of the effect of the RNAi-mediated suppression of dPerk on the transcript levels of *Relish* in the intestine of *pink1* mutants (asterisks, one-way ANOVA with Dunnett’s multiple comparison test, comparison of *pink1* mutant with control and each dPerk-RNAi line. Genotypes: **a** *w; NP3048Gal4/UASGFP;* + , **b** top: *w; elavGal4/UASGFP and* bottom*: w; NP3048Gal4/UASGFP* **c** Pink1: *pink1B9; mex1Gal4/+*; +, Pink1, dPerk-RNAi#1: *pink1B9; mex1Gal4/UAS dPerk RNAi#1* **d** control: *w; NP3048Gal4/* + *;* + , Pink1: *pink1B9; NP3048Gal4/* + ; +, Pink1, dPerk-RNAi#1: *pink1B9; NP3048Gal4/UAS dPerk RNAi#1*; +, Pink1, dPerk-RNAi#2: *pink1B9; NP3048Gal4/UAS dPerk RNAi#2*; +.

In addition to gut specific expression, the *NP3084*Gal4 driver also has some off-target expression in a small region of the brain [22]. The detection of dPerk levels when silencing dPerk by RNAi in the brain may be diluted if the driver only drives expression in a subset of neurons. Hence, we examined the expression of the *NP3084*Gal4 driver at the cellular level in the adult fly brain. We used *NP3084*Gal4 to drive GFP in adult flies and as expected we found that this driver induces GFP expression throughout the fly midgut (Fig. 8a).

We also analysed the cluster of PPL1 neurons in flies where GFP expression was driven by either *elav*Gal4, a pan-neuronal driver, or *NP3084*Gal4, the gut driver. As anticipated, we found that GFP is detected in the PPL1 and other clusters of neurons with *elav*Gal4 but not with *NP3084*Gal4 driver (Fig. 8b). This demonstrates that the *NP3084*Gal4 driver does not induce off-target expression in this subset of neurons that are lost in *pink1* mutant flies. Compared to the *NP3084*Gal4, another enterocyte driver, *mex1*Gal4 [23] was reported to have no off-target expression in other organs [22]. We also found that RNAi-mediated suppression of dPerk in the intestine, using *mex1*Gal4, rescued the loss of DA neurons in *pink1* mutant flies (Fig. 8c).

We have previously shown that the intestinal suppression of the NF-κB-like transcription factor, Relish, is neuroprotective in *pink1* mutant flies [11]. We next measured the expression of *relish* in the gut of *pink1* and *pink1, dPerk RNAi lines #1* and *#2*. We found an increase in the expression of *relish* in *pink1* mutants that was suppressed in one of the dPerk RNAi lines (Fig. 8d, e).

We therefore conclude that suppressing intestinal dysfunction by downregulating *dPerk* specifically in the midgut of *pink1* mutant flies is sufficient to rescue neurotoxicity.

## Discussion

We observed that sustained *dPerk* expression in the intestine led to loss of the Δψm and apoptotic cell death. During the first 24 h of sustained UPR activation, PERK signalling promotes the fission and fusion of mitochondria and enhances their function (reviewed in [24]). Following UPR activation for 24 h, mammalian cells eventually “give up the ghost”, and mitochondrial fragmentation leads to cellular suicide thorough apoptosis, a form of programmed cell death [25]. Our observations in the fly intestine extend these findings obtained in mammalian models by showing that sustained expression of *dPerk* causes loss of the Δψm. Previously, we showed that *dPerk* expression in flies reduces the levels of several mitochondrial components [26]; thus, we propose that loss of these components of the mitochondrial proteome causes a decline in mitochondrial function and loss of the Δψm.

We found that the suppression of *dPerk* in the enterocytes of *pink1* mutant flies prevented their apoptotic demise. Stress in enterocytes of adult *Drosophila* causes the proliferation of ISCs. This proliferation can be measured by counting PH3-positive cells. Upon cell division, each ISC produces one daughter cell that preserves the ISC fate and one that can develop into an enterocyte to replace damaged cells [21]. We observed that the suppression of dPerk by RNAi did not suppress the increase in PH3-positive cells found in *pink1* mutant flies (Fig. 6c). This signal presumably originates from stressed enterocytes to ISCs in *pink1* mutants and involves JNK and JAK-STAT as well as other signalling pathways [21] likely acting upstream of the apoptotic execution phase. Thus, even though the suppression of dPerk can prevent enterocyte cell death, this may not prevent cell-cell communication between enterocytes and ISCs in *pink1* mutants. It is also possible that the proliferation of ISCs is occurring in *pink1* mutants independently of enterocyte damage. The ISCs in *pink1* mutants are also likely to have mitochondrial damage. Mutations in *pink1* increases the levels of mitochondrial reactive oxygen species (ROS) in flies [27]. ROS can control the proliferation of ISCs (reviewed in [28]) and therefore act as a cell autonomous mechanism that triggers their proliferation, independent of damaged enterocytes.

Both the intestinal suppression of Relish (our previous work [11]) and dPerk (this study), using a suite of three independent enterocyte Gal4 drivers, *NP3084*Gal4, *NP1*Gal4 [11] and *mex1*Gal4, confer neuroprotection to *pink*1 mutant flies. To understand the relationship between the regulation of *dPerk* and *relish* signalling, we measured the expression of *relish* following the downregulation of *dPerk* in *pink1* mutant flies. We found that downregulating *dPerk* using one of the two *dPerk* RNAi lines significantly lowered expression levels of *relish* in *pink1* mutants. These findings indicate that dPerk may alter intestinal homeostasis by inhibiting an inflammatory response within the gut. Decreasing *dPerk* levels in the intestine of *pink1* mutants might improve mitochondrial function (as observed by measuring Δψm, Fig. 5), by decreasing the amount of DNA released by mitochondria and dampening the inflammatory response. However, it is still unclear first, why the transcript levels of *relish* are increased in the intestine of *pink1* mutant flies and second, why suppressing *dPerk* reduces *relish* transcript levels.

The downregulation of *relish* in the intestine of *pink1* mutant flies was not observed with dPerkRNAi#2 (Fig. 8d). However, we noted that the suppression of *dPerk* levels using RNAi#2 is not as efficient as those with RNA#1 line (Fig. 7d). It is therefore possible that the lack of the downregulation of *relish*, conferred by the expression of dPerk RNAi#2 in the intestine of *pink1* mutants, is a consequence of the moderate loss of *dPerk* expression in this line.

Our results build on prior observations [11] to show that a gut-brain pathway causes neurotoxicity linked to mitochondrial dysfunction in *Drosophila*. In mice, this gut-brain route involves the vagus nerve [5]. In our model, it is unclear how mitochondrial toxicity is passed from the gut to the CNS. We reason that this transmission is indirect, as there are currently no known connections between the gut and DA neurons. Nevertheless, there are connections between the CNS and the gut that involve neurons with cell bodies in the posterior segments of the abdominal ganglion of the ventral nerve cord that send axons to the posterior portion of the midgut. These connections are relayed to the CNS by insulin-producing neurons [29]. It is possible that these insulin-producing neurons form a “wired” route to transmit mitochondrial toxicity from the gut to the brain in *pink1* mutant flies. An alternate indirect mechanism through which mitochondrial toxicity can be transmitted from the gut to the brain could involve a “wireless” mechanism. Crewe and colleagues [30] reported that mammalian cells can communicate mitochondrial stress via a “wireless” humoral mechanism. This mechanism involves the release of mitochondrial particles (small extracellular vesicles) into the circulation and the transport of these particles between the fat body and heart. Similar mechanisms may occur in flies, where mitochondrial vesicles communicate stress between the intestine and the brain. Our study using fruit flies highlights the importance of the gut-brain axis in the onset and progression of PD. This supports Hippocrates’s 2000-year-old claim that “all disease begins in the gut”.

## Methods

### Genetics and *Drosophila* strains

Fly stocks and crosses (unless otherwise stated) were maintained on standard cornmeal agar media at 25 °C. The following strains were used: *pink1*^*B9*^ as previously described (Tufi et. al., 2014), dPerk RNAi lines (RNAi#1, ID:16427; RNAi#2, ID: 110278) (Vienna Drosophila RNAi Centre, Vienna, Austria), *w; P{GawB}NP3084;* + (KSC_113094), *w; UAS dPerk; +*,*w; UAS dPerk-KD;* + as previously described [26], *w; tubP-Gal80*^*ts*^*/TM2* (Bloomington 7017) and w; *P{mex1-Gal4}* (Bloomington 91368). All crosses involving UAS dPerk and UAS dPerk-KD were kept at 18 °C, and progenies with selected genotypes were kept at 25 °C until assayed. All crosses involving Gal80^ts^ were kept at 18 °C, and the hatched progenies with selected genotypes were kept at 29 °C until assayed. All the experiments on adult flies were performed using males.

### Analysis of faecal output and the T.U.R.D. assay

To aid the visualisation of fly excreta, 0.5% Brilliant Blue FCF (80717, Sigma‒Aldrich) was added to standard cornmeal agar media. The dye-containing food was offered ad libitum as a wedge of solid food placed on a Petri dish, and five 10-day-old males were allowed to excrete on the clear flat plastic surface of the dish for 20 hours at 25 °C. A total of twenty-five flies per genotype were examined. At the end of the experiment, the flies and food were removed from the Petri dishes, and the dishes containing fly deposits were scanned with a transparency scanner (Epson Perfection, V750 Pro). Images were cropped and prepared for analysis using Adobe Photoshop 2022 (23.4.1). Images of fly faecal deposits were analysed using T.U.R.D. software, a tool designed to assess the colour and morphology of excreta produced by flies fed on a dye-supplemented diet [31].

### Locomotor assays

Twenty-five-day-old males were individually loaded into Drosophila Activity Monitors (DAM5) within 8 × 65-mm^2^ glass Pyrex tubes (Trikinetics, Waltham) containing normal fly food. The flies were maintained at 25 °C on a 12/12-h light-dark cycle for at least 8 days. Sleep and activity data were analysed using the Sleep and Circadian Analysis MATLAB Program (SCAMP) developed by the Griffith laboratory [32]. Analyses were performed for 7 days starting at the first Zeitgeber time (ZT0) to allow acclimation. At least 40 flies of each genotype were used. Flies with rhythmic index scores < 1 were removed from the analyses.

### Lifespan analysis and eclosion

Groups of 12 newly eclosed males of each genotype were placed into separate vials containing food and maintained at 25 °C (or at 29 °C for Gal80^ts^ experiments). The flies were transferred to vials containing fresh food every 2 to 3 days, and the number of dead flies was recorded. The data are presented as Kaplan‒Meier survival curves, and significance was determined by log-rank tests. For eclosion assays, crosses were set in multiple vials with 10 virgins and 4 males for all conditions and allowed to lay for 2 days and the number of empty and total pupae scored at the end of eclosion. The data are presented as percentages, and significance was determined by using the Chi-squared test.

### Microscopy-based assessment of mitochondrial function and morphology

The Δψm in the fly gut was measured as previously described [33]. Briefly, adult fly midguts were incubated for 40 minutes at room temperature with 25 nM TMRM in 1× Hank’s Balanced Salt Solution (HBSS) (14175-053, Gibco). TMRM was used in redistribution mode to assess the Δψm; therefore, a reduction in the TMRM fluorescence intensity indicated mitochondrial depolarization. Confocal images were acquired using a Zeiss LSM 880 or 980 confocal microscope equipped with a 20× objective. The illumination intensity was kept at a minimum (at 0.1–0.2% of the laser output) to avoid phototoxicity, and a pinhole was used to obtain a 1 μm thick optical slice. The fluorescence intensity of TMRM was quantified at an excitation wavelength of 565 nm and emission wavelength greater than 580 nm. A single Z-stack per gut was acquired, and 8 10–20 μm^2^ ROIs were selected. The mean maximal fluorescence intensity in these ROIs was measured.

### Immunofluorescence and confocal microscopy

To image the intestine, guts were dissected from live flies in ice-cold PBS and fixed in 4% PFA (for anti-HA, anti-PH3, anti-TH and anti-GFP antibody incubation) or heptane-methanol fixative (for anti-Dcp1 antibody incubation) for 30 minutes and blocked overnight in 10% normal goat serum PBS/0.5% Triton. The samples were then incubated with primary antibodies (anti-cleaved *Drosophila* Dcp-1 (CST, cat no. 9678, 1:100), anti-HA (Roche Applied, no. 11583816001, 1:1000) and anti-PH3 (mix 1-1 of CST, cat no. 9701 (Ser10) and cat no. 9713 (Ser28), 1:100), anti-GFP (ab13970, 1:1000) and anti-TH (Immunostar, 22941, 1:50) at 4 °C overnight and then with secondary antibodies (Alexa Flour™ 488-conjugated F(ab’)2 fragment of goat anti-rabbit IgG (H + L) (Invitrogen, A11070), Alexa Flour™ 488-conjugated goat anti-chicken IgG (H + L) (ab 150169) or Alexa Flour™ 568-conjugated goat anti-mouse IgG (H + L) (Invitrogen, A11031) and Hoechst 33342 (1:500, Invitrogen, H3570) diluted 1:500 for 2 hours at room temperature.

### Analysis of DA neurons

Brains from 30-day-old flies were dissected and stained with anti-TH antibody (Immumostar, no. 22941, 1:50) as previously described [34]. The brains were placed in PBS + 0.1% Triton in a coverslip clamp chamber (ALA Scientific Instruments) using a harp composed of platinum wire and nylon string and imaged by confocal microscopy. The numbers of TH-positive neurons in the PPL1 cluster in each brain hemisphere were determined.

### RNA extraction and reverse transcription quantitative real-time PCR

Total RNA was extracted from 25 heads or 15–20 freshly dissected guts using TRIzol (Ambion) and quantified by spectrophotometric analysis (Nanodrop, Thermo Scientific). RT‒qPCR was performed with a real-time cycler (Applied Biosystems 75000, Fast Real-Time PCR Systems) using the SensiFAST SYBR Lo-ROX One-Step Kit (Bioline). The fold change in expression was calculated using the comparative Ct method [35]. For RT‒qPCR, we measured the coefficient of variance (CV) of the technical replicates, and any samples with a CV > 3% was excluded from statistical analysis. The gene-specific primers were Dm_PEK_2_SG QuantiTect Primer Assay (Qiagen, QT00968618), and *Relish* (forward, 5′-CATCAGGAGACAGAGCGTGA-3′; reverse, 5′-CCGACTTGCGGTTATTGATT-3′) and rp49 (forward, 5′-TGTCCTTCCAGCTTCAAGATGACCATC-3′; reverse, 5′-CTTGGGCTTGCGCCATTTGTG-3′) (Sigma‒Aldrich).

### Statistical analyses

Statistical analyses were performed using GraphPad Prism (www.graphpad.com). The data are presented as the mean values, and the error bars indicate ± sem. In the violin plots, the solid line represents the median, while the dashed lines represent the quartiles. The individual data points in the figures correspond to biological replicates. The number of biological replicates per experimental variable (n) is indicated in either the figure or respective figure legend. For all statistical analyses, the Shapiro–Wilk test was used to test if the data followed a normal distribution. Based on normality test results, parametric or nonparametric analysis was used. Samples were excluded from the analysis based on the Prism outlier test, ROUT, Q = 1%. Blinding was not performed. Significance is indicated as * for *P* < 0.05, ** for *P* < 0.01, *** for *P* < 0.001, **** for *P* < 0.0001 and NS for *P* ≥ 0.05.

### Digital image processing

Fluorescence and transmission electron microscope images were acquired as uncompressed bitmapped digital data (TIFF format) and processed using Adobe Photoshop, employing established scientific imaging workflows.

## Supplementary information

Author checklist

## Data Availability

The authors confirm that the data supporting the findings of this study are available within the article.
